# Hospitals for the Mentally Deficient

**Published:** 1907-07-13

**Authors:** 


### HOSPITALS FOR THE MENTALLY DEFICIENT.

### The Morningside Centenary.

We have always felt that the term "asylum" should be abandoned, so far as institutions for the insane are concerned, in favour of "hospital." Unfortunately the lunatic is regarded as a waste product, and although he is now frequently housed in something like a palace, with palatial grounds and extensive park lands, his movements and condition are matters which seldom or never trouble the minds of the editors of great newspapers or of the people at large. Indeed, there is an undercurrent of feeling which may produce very striking changes in the next generation in favour of the view that it is hardly justifiable to spend huge sums of money annually upon members of the race who, for all practical purposes, have ceased to exist in a conscious and mental state. The managers of our hospitals for the insane are therefore wise to seek every opportunity to attract public interest in their work, population, and establishments. Much more than local interest attaches to the centenary celebrations of the Royal Edinburgh Asylum on Friday, July 12. This establishment originated in 1792 through the death of the poet Fergusson, the precursor of Burns. Fergusson was an erratic genius, whose sprightly gaiety made him much sought after as a companion, and in his day, though he died at 23, he was the life of the company of the Auld Reekie, a noted club in Edinburgh in the eighteenth century. Fergusson's head was severely injured by a fall, which aggravated earlier symptoms of mental aberration, and led to his confinement in the old Darien House, where he died. This was the only public asylum in Edinburgh, and the removal and death there of so noted a character aroused public sentiment and led ultimately to the founding of a mental hospital by Dr. Andrew Duncan, President of the College of Physicians, Edinburgh, in 1792. This effort, though taken up by public men and public bodies, failed, but within 15 years a grant of £2,000 was obtained from estates forfeited in the rebellions of 1715 and 1745. A year later a royal charter was obtained, and the foundation-stone of the East House of the new mental hospital was laid in 1809, the hospital itself being opened four years later. West House was added in 1837, and in 1840 Dr. Mackinnon was appointed the first physician superintendent. In 1841 the hospital was named the Royal Edinburgh Asylum, and Queen Victoria became its patron. Dr. Skae became physician superintendent in 1846, and in 1873 was succeeded by Dr. Clouston, who is deservedly and widely known as one of the greatest of alienists. Under Dr. Clouston's *régime* Morningside has been developed, new and original methods have been introduced, and there is no place where more matter for consideration and instruction is to be met with by those who have eyes to see. It is only right and proper that this hospital for the insane should celebrate its centenary worthily. We look forward to even greater developments and added authority for a foundation full of interest to the historian and of practical instruction for the expert.

### Some Burning Topics.

The continuous increase in local rates is causing a discussion amongst some of the more thoughtful members of county councils and other bodies as to the present high rate of expenditure in the accommodation and treatment of the insane. The question is being asked as to how far it is justifiable, in view of the great amount of distress which prevails among many of the poor who are healthy and well in body and mind, to incur avoidable expenditure in various directions in order to surround the hopelessly decrepit, devoid of all mental apprehension, not only with every comfort but with many luxuries of various kinds. There is the further point that, in some lunatic asylums, large sums have recently been expended on tubercular wards, where the chronic lunatic affected with tuberculosis is treated at great cost and in many cases restored to robust health. In the ordinary course, as things were formerly, such cases would not continue to live for more than a few years, if so long, whereas they may now be restored to a condition of physical strength which may keep them alive for 20 or 30 years longer, without any benefit to themselves or to anyone else. Is this policy, and the large expenditure involved, justifiable? If so, on what grounds, and to what extent? At an asylum we recently inspected the authorities have erected tubercular wards apart from the main building, and have moved all phthisical cases from the asylum dormitories into the special wards. In this one asylum the consequent reduction in the average mortality of the patients will, it is estimated, increase the cost to the ratepayers, so far as the 50 patients treated in the special wards are concerned, by no less a sum than £145,000. If this policy is to be followed throughout the country, the additional burden cast upon the ratepayers must amount to several millions sterling. If it is right to expend all this money on the unfortunates of our race who lack the mental strength to maintain themselves, it is a great reflection upon modern methods that the healthy are permitted to be infected by the tuberculous when the former happen to be members of a family, and not lunatics in a public asylum. It would be dangerous doctrine to refuse a lunatic such of the advantages of modern therapeutic methods as are equally to be obtained by those not under restraint, such as, for instance, the operative treatment of malignant disease; but it does seem a waste of time and money to grant him actual preferential treatment which ordinary members of the community do not share. The present Government seems very ready to appoint Royal Commissions, and we hold that there is pressing necessity, in the best interests of the public at large, for a Royal Commission to inquire into the vital questions which underlie the points raised.

